# The Augmenting Effects of Desolvation and Conformational Energy Terms on the Predictions of Docking Programs against mPGES-1

**DOI:** 10.1371/journal.pone.0134472

**Published:** 2015-08-25

**Authors:** Ashish Gupta, Neha Chaudhary, Kumar Reddy Kakularam, Reddanna Pallu, Aparoy Polamarasetty

**Affiliations:** 1 Centre for Computational Biology and Bioinformatics, School of Life Sciences, Central University of Himachal Pradesh, Dharamshala, Himachal Pradesh– 176215, India; 2 Department of Animal Biology, School of Life Sciences, University of Hyderabad, Hyderabad, Telangana– 500046, India; University of Akron, UNITED STATES

## Abstract

In this study we introduce a rescoring method to improve the accuracy of docking programs against mPGES-1. The rescoring method developed is a result of extensive computational study in which different scoring functions and molecular descriptors were combined to develop consensus and rescoring methods. 127 mPGES-1 inhibitors were collected from literature and were segregated into training and external test sets. Docking of the 27 training set compounds was carried out using default settings in AutoDock Vina, AutoDock, DOCK6 and GOLD programs. The programs showed low to moderate correlation with the experimental activities. In order to introduce the contributions of desolvation penalty and conformation energy of the inhibitors various molecular descriptors were calculated. Later, rescoring method was developed as empirical sum of normalised values of docking scores, LogP and Nrotb. The results clearly indicated that LogP and Nrotb recuperate the predictions of these docking programs. Further the efficiency of the rescoring method was validated using 100 test set compounds. The accurate prediction of binding affinities for analogues of the same compounds is a major challenge for many of the existing docking programs; in the present study the high correlation obtained for experimental and predicted pIC_50_ values for the test set compounds validates the efficiency of the scoring method.

## Introduction

Microsomal prostaglandin E synthase-1 (mPGES-1) belongs to the membrane-associated proteins involved in eicosanoid and glutathione metabolism (MAPEG) super family [1]. It is the terminal enzyme in the metabolism of arachidonic acid (AA) via the cyclooxygenase (COX) pathway (particularly COX-2), responsible for the conversion of prostaglandin H_2_ (PGH_2_) to a more stable product prostaglandin E_2_ (PGE_2_). As PGE_2_ is a key mediator of pain and inflammation [2], the enhanced mPGES-1 expression is associated with many pathological conditions in humans; including myositis [3], rheumatoid arthritis [4], osteoarthritis [5], inflammatory bowel disease [6], cancer [7, 8], atherosclerosis [9], and Alzheimer’s disease [10]. So, efforts are being made by several pharma companies for the development of anti-inflammatory drugs, targeting mPGES-1.

Recently Zhan *et al*., generated the 3D structure of human mPGES-1 by employing homology modelling approaches (11). Further, they applied molecular docking and molecular dynamics simulations to get detailed insights into the substrate binding domain (SBD) of mPGES-1 protein. Koeberle and collaborators [11] have recently identified pirinxic acid derivatives as potent mPGES-1 inhibitors, with IC_50_ of 1.3 μM. Hamza *et al*. [12] have also developed a series of novel mPGES-1 inhibitors by employing a combination of large-scale structure-based virtual screening, flexible docking, molecular dynamics simulations and binding free energy calculations. They identified (Z)-5-benzylidene-2-iminothiazolidin-4-one as a novel scaffold for further rational design and discovery of new mPGES-1 inhibitors. In one of the recent reports, Arhancet *et al*. [13] described the discovery of PF-4693627 as a potent mPGES-1 inhibitor, by employing SAR and lead optimisation studies, for the potential treatment of inflammation. This compound had improved pharmacokinetic profile with potent inhibition of mPGES, both *in vitro* and *in vivo*. The application of computational studies in drug discovery projects is very challenging. Simple docking algorithms are not accurate enough for *in silico* activity predictions, whereas computationally expensive/efficient simulation methods require great expertise and computational facilities. Hence there is a need to develop accurate and computationally inexpensive methods for prediction of activity against mPGES-1. Molecular docking is a key tool in structural molecular biology and computer-assisted drug design. During the last three decades molecular docking has emerged as a key tool in structure-based drug discovery. Molecular docking helps us to understand and predict molecular recognition, both structurally (predicting binding modes), and energetically (predicting binding affinity) between entities of interest. Docking has two main constituents, a scoring function and a search method. Scoring functions segregate the various conformations generated on the basis of the most effective binding interactions between the ligand and the protein [14]. It is a known fact that docking forms a good tool for predicting the different poses or conformations in which the ligand binds to the protein. The accurate prediction of the relative binding affinities (RBAs), however, still remains a challenging task [14–16]. This is due to the fact that a single scoring function cannot hold well under all circumstances. In order to get insights into this problem Warren *et al*. [15] performed thorough studies with a large and diverse set of receptors and ligands by using different methodologies. When the results were analysed they found very weak correlation between the measured and calculated binding affinities. The scoring functions of most of these docking programs are too general i.e. they are not target specific. Drug discovery researchers started developing tuned/consensus scoring functions which can increase the accuracy of *in silico* predictions [17–23]. Various studies have shown that the application of scoring functions together with other scoring functions or molecular descriptors can improve the performance significantly. In the present study we developed a scoring methodology specific to mPGES-1 which may be useful for more accurate prediction of binding affinities and thus facilitating the medicinal chemistry projects to identify and discover more potent inhibitors for mPGES-1.

## Material and Methods

### Preparation of Ligands

**Fig 1:**
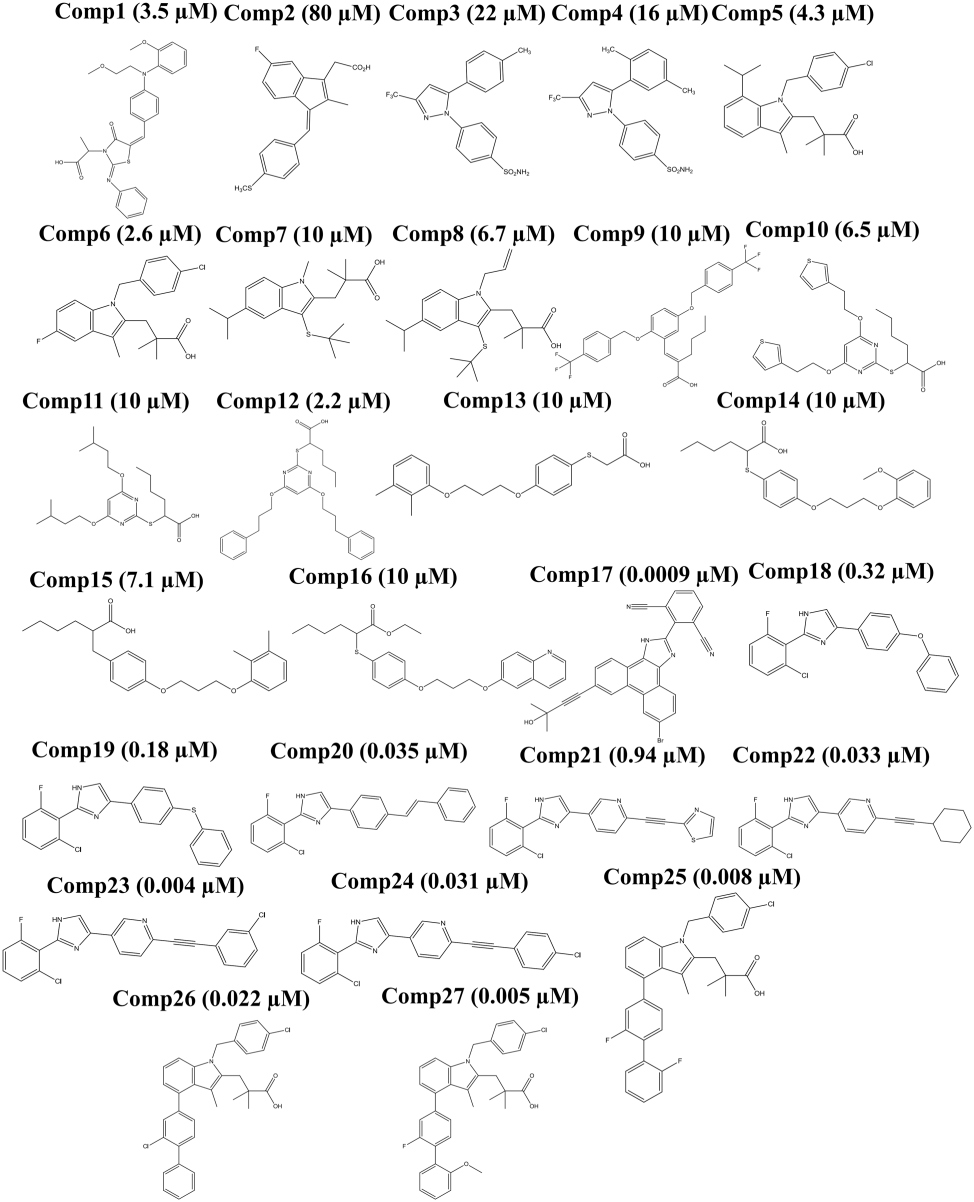
Structure of training set compounds.

For this study 127 inhibitors of mPGES-1 were selected randomly from literature and BRENDA [24] database. All the structures were prepared in Accelrys Draw and optimized initially using HF method in R.E.D server [25–29] and further optimized using DFT based method i.e. B3LYP/6-31G(d) [30, 31] in Gaussian09 [29] to get the lowest energy conformations. The lowest energy conformations from Gaussian were further used for docking. The dataset was further segregated into training set (27 compounds) (Fig 1) and external test set (100 compounds) (Fig A,B,C in S1 File).

### Docking

The prepared ligand structures were then docked into the mPGES-1 binding site using default procedure implemented in AutoDock Vina [32], AutoDock [33], DOCK6 [34] and GOLD [35] programs. The binding site of mPGES-1 was defined as was described earlier by Prage *et al*. [36], and Jakobsson *et al*. [37]. All the input and output files for the docking programs used can be found in the supplementary material.

### Auto Dock Vina

Auto Dock Vina is based on Lamarckian genetic algorithm and empirical binding free energy force field, assuring its enhanced performance and accuracy. After the preparation of ligands and target protein and selecting the binding site residues in mPGES-1 the grid was placed in the centroid of the selected residues. Then docking was performed using the default settings of Auto Dock Vina. AutoDock Vina generates a maximum of 20 conformations for a single ligand and the same were used in the present study. The scoring function of Vina, has advantages of both knowledge-based potentials and empirical scoring functions. It extracts information both from the conformational preferences of the receptor-ligand complexes and the experimental affinity measurements [32].

### AutoDock

AutoDock is an automated procedure for calculating the interaction of ligands with biomacromolecular drug targets. In the present study AutoDock 4.0 was used. AutoDock employs Lamarckian genetic algorithm and empirical free energy scoring function to generate the binding modes of ligand within the protein active site [33]. The target protein and ligands were prepared for docking calculation. The coordinates of the active site of the protein was used for generation of grid file. Docking was performed and 200 conformations were generated for every ligand.

### Dock

Dock predicts the correct binding mode of small molecule in the binding site of protein, and the corresponding binding energy using anchor and grow algorithm. In the present study DOCK6 was used. For docking the box was generated using the GSH binding site coordinates of mPGES-1; grid was computed using grid parameter file and flexible ligand docking was performed using default parameters and for each ligand 200 conformations were generated. Grid score of DOCK6 was used in the present study. The scoring in DOCK is based on the non-bonded terms of molecular mechanic force field [38].

### GOLD

GOLD (Genetic Optimization of Ligand Docking), a genetic algorithm based docking program [36], was also used to perform the docking calculations. During docking, the default algorithm speed was selected and the ligand binding site in mPGES-1 was defined. The number of poses for each inhibitor was set to 200, and early termination was allowed if the top three bound conformations of a ligand were within 1.5 Å RMSD. Higher Goldscore implies better result. The Goldscore, Chemscore [39] and Astex Statistical Potential (ASP) score [40] are the scoring methods available in GOLD.

### Consensus Scoring

Consensus scoring is a method in which the binding affinities of ligands for a drug target are predicted by using more than one scoring method [41]. In this study, a consensus scoring approach was applied as an average of scores of AutoDock Vina and GOLD (Chem score, Asp score and GOLD fitness score). As the scoring functions of AutoDock Vina and GOLD programs are in different range, prior to mean calculation and other statistical operations on the docking scores, data normalization was performed to bring all the scores in a notionally common scale from 0 to 1 (Table 1). Data normalization was performed using the formula: (For positive scores)

$$Normalized\;score=\frac{\left(x-\text{min}\right)}{\left(\text{max}-\text{min}\right)}$$

(For negative scores)

$$Normalized\;score=1-\frac{\left(x-\text{min}\right)}{\left(\text{max}-\text{min}\right)}$$

Where x = corresponding score, max = maximum score and min = minimum score of the dataset

**Table 1:** Normalized scores of various docking programs and molecular descriptors.

Compounds	pIC_50_	Goldscores	Chem Score	AutoDock score	Auto Dock Vina Score	DOCK6 Grid Score	ASP Score	LogP	TPSA	Vol	Nrotb	Consensus score
1	5.46	0.19	0.33	0.12	0.55	0.67	0.48	0.47	0.95	0.88	0.6	1.54
2	4.10	0.22	0.47	0.74	0.75	0.12	0.16	0.04	0.13	0.00	0.2	1.60
3	4.66	0.28	0.54	0.26	0.70	0.01	0.24	0.01	0.73	0.00	0.2	1.76
4	4.80	0.31	0.50	0.40	0.75	0.07	0.22	0.08	0.73	0.08	0.2	1.78
5	5.37	0.00	0.23	0.80	0.50	0.08	0.00	0.57	0.20	0.38	0.33	0.73
6	5.59	0.40	0.16	1.00	0.60	0.13	0.24	0.24	0.20	0.16	0.27	1.40
7	5.00	0.08	0.15	0.79	0.35	0.20	0.01	0.46	0.20	0.30	0.33	0.59
8	5.17	0.21	0.00	0.55	0.15	0.18	0.06	0.58	0.20	0.44	0.47	0.42
9	5.00	0.44	0.52	0.20	0.90	0.61	1.00	0.90	0.40	0.82	0.8	2.86
10	5.19	1.00	0.12	0.27	0.45	0.80	0.57	0.45	0.78	0.58	0.87	2.14
11	5.00	0.60	0.15	0.04	0.00	0.56	0.33	0.55	0.78	0.46	0.87	1.08
12	5.66	0.91	0.36	0.05	0.25	1.00	0.75	0.79	0.78	0.84	1	2.27
13	5.00	0.34	0.56	0.51	0.25	0.30	0.28	0.16	0.40	0.10	0.53	1.43
14	5.00	0.46	0.59	0.38	0.65	0.68	0.49	0.29	0.54	0.40	0.8	2.18
15	5.15	0.51	0.84	0.39	0.00	0.46	0.37	0.53	0.40	0.43	0.73	1.72
16	5.00	0.51	1.00	0.00	-0.05	0.91	0.68	0.54	0.43	0.64	0.87	2.14
17	9.05	0.57	0.65	0.34	0.80	0.21	0.21	0.51	1.00	0.51	0	2.23
18	6.49	0.37	0.35	0.32	0.85	0.00	0.50	0.48	0.14	0.04	0.2	2.07
19	6.74	0.24	0.42	0.36	1.00	0.01	0.51	0.52	0.00	0.09	0.2	2.17
20	7.46	0.24	0.77	0.28	0.75	0.19	0.34	0.63	0.00	0.13	0.2	2.10
21	6.03	0.59	0.63	0.15	0.55	0.04	0.51	0.00	0.38	0.02	0.07	2.28
22	7.48	0.54	0.67	0.17	0.60	0.00	0.41	0.44	0.19	0.18	0.07	2.22
23	8.40	0.61	0.81	0.13	0.80	0.06	0.65	0.36	0.19	0.15	0.07	2.87
24	7.51	0.54	0.73	0.08	0.60	0.05	0.60	0.37	0.19	0.15	0.07	2.47
25	7.66	0.40	0.62	0.78	1.00	0.32	0.77	1.00	0.20	0.90	0.4	2.79
26	8.30	0.40	0.76	0.77	0.80	0.51	0.73	0.96	0.20	0.92	0.47	2.70
27	8.10	0.41	0.74	0.80	0.90	0.44	0.75	0.97	0.34	1.00	0.4	2.80

After data normalization and calculation of consensus score, correlation coefficient between the activity (pIC_50_) and the consensus score was calculated. It was compared with correlation coefficient of all docking programs.

### Receptor Specific Tuning/Rescoring Method

For the design of inhibitors, the detailed knowledge of thermodynamics of ligand binding is very important. Upon binding of a ligand to its drug target, the change in Gibbs free energy, known as free energy of binding (ΔG_bind_), determines the ligand’s binding affinity [42].

As ΔG_bind_ is dependent on the change in enthalpy and entropy, optimizing these factors can improve affinity of ligand [43]. The protein—ligand interactions contribute for the enthalpic component while entropy is primarily attributed to the hydrophobic effect and desolvation penalty, which can be explained in terms of molecular descriptors LogP, topological polar surface area (TPSA) and volume of the inhibitor (Vol) [44, 45]. It is important to note that protein-ligand binding always takes place in aqueous environment. During the binding of ligand to the protein a series of events will take place, i.e. desolvation of ligand and protein, conformational changes, and formation of intermolecular interactions [46, 47].

Hence, in the present study, some molecular descriptors were also incorporated with the docking scores for the accurate prediction of binding affinities of ligands towards mPGES-1. The molecular descriptors considered in the studies were LogP, TPSA, Vol and number of rotatable bonds (Nrotb). The reason for including these molecular descriptors in the study was to introduce the concept of desolvation energy penalty and conformational free energy changes occurring when a ligand binds to a protein. For the prediction of LogP, TPSA, Vol and Nrotb, a web based server named Molinspiration was used.

The total free energy of binding can be expressed as:

$$-\Delta G_{bind}=\Delta G_{complex}-(\Delta G_{protein}+\Delta G_{ligand})=\Delta G_{MM}+\Delta G_{sol}-T\Delta S$$

Where, ΔG_MM_ = molecular mechanics free energy;

ΔG_sol_ = solvation free energy

TΔS = entropy contribution

The conformational energy penalty is critical for accurate estimation of free energy of binding (ΔG_bind_) of inhibitors [48]. Siebel *et al*. [49, 50] and Liljefors *et al*.[51] found that with every 1.4 kcal/mol increase in conformational energy of the bioactive conformation, there is decrease in binding affinity by a factor of 10. The conformational energy that is required for the ligand to adopt its bioactive conformation is crucial in understanding structure-activity studies [51, 52] and is critical in computer-aided ligand design [49]. In the present study, number of rotatable bonds (Nrotb) in the ligands was considered as descriptor of ΔG_conf_. The normalised values of LogP and Nrotb were calculated and added empirically to the normalised scores of docking programs to assess the effects of these molecular descriptor terms on their predictions.

### Validation

The most accurate rescoring methods and consensus scores were further validated using external test set of 100 compounds. Regression analysis was performed on the training set of 27 compounds to identify the weights of each individual component in consensus score and rescore. The weights were further used to predict/ calculate the affinity of test set compounds. Statistical analysis of the predictions was performed using SPSS statistical software. The rescore was tested for its ability to predict compounds as active and inactive using the training set. The training set contained 8 active, 9 inactive and 10 moderately active compounds. Efficiency of the rescores was measured in terms of the number of active compounds in the top 8 and the number of inactive compounds in the bottom 9.

## Results and Discussion

**Fig 2:**
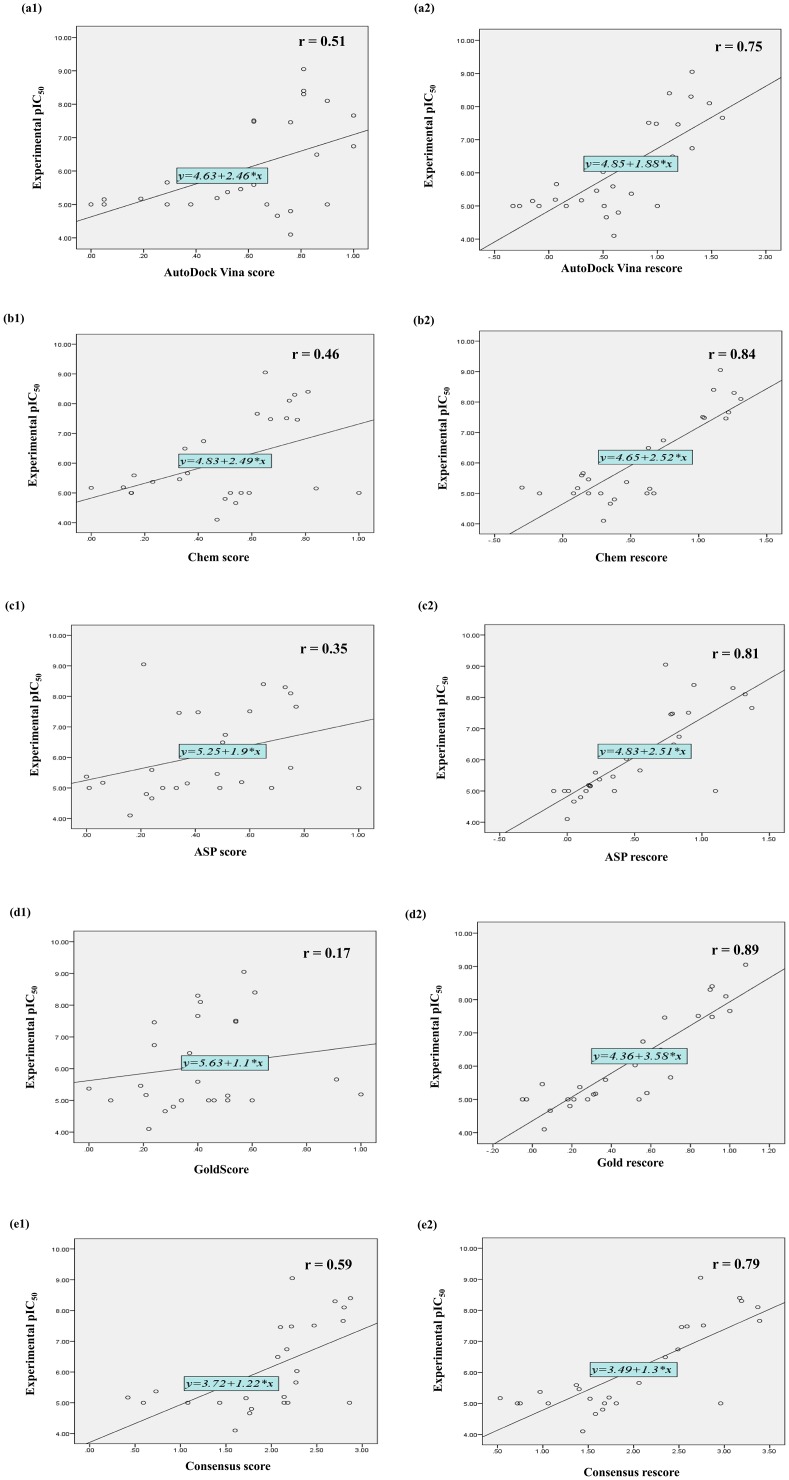
Scatter plots showing coefficient of correlation (r) and best line of fit for training set compounds (a1 and a2 = AutoDock Vina score and AutoDock Vina rescore; b1 and b2 = Chem score and Chem rescore; c1 and c2 = ASP score and ASP rescore; d1 and d2 = Goldscore and Gold rescore; and e1 and e2 = Consensus score and Consensus rescore respectively).

The scores from various docking programs and molecular descriptors were considered as the focus is on the development of a reliable consensus/rescoring method for *in silico* mPGES-1 activity prediction. The data from various programs was normalized to a common range of 0 to 1. The correlation coefficient (r) of scores of each individual program and mPGES-1 inhibition activity were calculated. Out of the four programs used, AutoDock Vina score exhibited most significant correlation (r = 0.51) with the activity (pIC_50_) of the training set compounds, followed by Chem score (r = 0.46) and ASP score (r = 0.36) (both obtained from GOLD program), while the GOLD fitness score showed a correlation of 0.17. The average of the above mentioned scores was considered as the consensus score. Consensus score showed positive correlation (r = 0.59) with the activity of the training set compounds, better than the entire individual scores (Fig 2).

The correlation of LogP TPSA, Vol and Nrotb with the activity were analysed (Table 2), and it was found that out of the descriptors considered for ΔG_solv_, LogP showed highest positive correlation with the activity of the compounds and rotatable bonds showed negative correlation. The negative correlation of Nrotb was in agreement to previous reports which suggest flexibility of ligand contributes in the form of conformational free energy. So in the present study, LogP and Nrotb were considered as molecular descriptors of ΔG_solv_ and ΔG_conf_ respectively.

**Table 2:** Correlation of normalized docking scores and molecular descriptors with pIC_50_.

S No	Scores/ Molecular Descriptor	Correlation with pIC_50_
1.	AutoDock Vina score	0.51
2.	Chem score	0.46
3.	ASP score	0.35
4.	GOLD Fitness score	0.17
5.	AutoDock score	0.02
6.	DOCK6 score	-0.23
7.	Consensus score (Avg of 1 to 4)	0.59
8.	LogP	0.45
9.	Volume of the inhibitor (Vol)	0.20
10.	TPSA	-0.21
11.	No. of rotatable bonds	-0.48

The major component in free energy of the ligand is the interaction energy between protein and ligand, ΔG_inter_. The docking scores were considered as a measure of ΔG_inter_. For the rescoring method the effective binding free energy of each ligand was considered as an empirical summation of normalised docking scores, LogP and Nrotb.

The rescoring function was calculated as:

Re score=Docking score+LogP−Nrotb

The correlation of the rescores with activity of the compounds was calculated (Table 3) and the results showed significant improvement in the predictions of various docking programs. The correlation of AutoDock Vina scores increased drastically from 0.51 to 0.75, with the inclusion of molecular descriptors LogP and Nrotb. Most significant improvement was seen in the case of Goldscore which increased to 0.89 from 0.17 with the molecular descriptor terms. The consensus score also improved from 0.59 to 0.79. Overall the scores showed that there was additive effect which caused improvement in the correlation of all the scores considered. These results further confirmed the effectiveness of LogP and Nrotb as molecular descriptors of ΔG_solv_ and ΔG_conf_ respectively.

**Table 3:** Pearson and Spearman correlation between scores and rescores of docking programs with experimental values (pIC_50_) and sigma 2-tailed tests for the training set.

		pIC_50_	AutoDock Vina rescore	Chem rescore	ASP rescore	GOLD rescore	Consensus rescore
**pIC**_**50**_	Pearson Correlation	1	.75**	.84**	.81**	.89**	.79**
	Spearman Correlation	1.00	.67**	.69**	.81**	.88**	.71**

** denotes that the correlation is significant at 99% confidence level

The predictions of the training set compounds were further analysed. The 27 compounds taken for training set were classified into 8 active (IC_50_ ≤ 100nM), 10 moderately active (100nM ≥ IC_50_ ≤ 10 μM) and 9 inactive molecules (IC_50_ ≥ 10 μM). These compounds were then taken as reference for validating the efficiency of the docking scores and rescores in differentiating active and inactive molecules from a pool of compounds. Firstly the individual docking scores (AutoDock Vina, Chemscore, ASP score, Goldscore and Consensus scores) were analysed to get insights into the efficiency of the docking programs in predicting the binding affinities and later the same operation was performed on the developed rescores.

Docking scores and rescores of the training set compounds were analysed to identify the compounds occupying the top 8 and bottom 9 places. In the normalised AutoDock Vina score, it was observed that the active compounds were having scores > 0.8 whereas the inactive compounds were showing scores < 0.5. On this basis the cut-off values were designated to the active (scores > 0.8) and inactive compounds (scores < 0.5) for AutoDock Vina scores and further analysis was performed on the basis of these cut-off values. Similarly, in the case of Chem score top 8 compounds were having scores > 0.67, while the bottom 9 compounds showed Scores < 0.36, in case of ASP scores, active compounds exhibited scores > 0.6, whereas inactive compounds had scores < 0.28; in case of Goldscores the top 8 compounds showed scores > 0.54 and the bottom nine (inactives) had scores < 0.31, so the same cut-off value were assigned to characterize training set compounds as active and inactive. Active compounds of the consensus score were the ones having score > 1.86 and compounds having scores < 1.35 were considered as inactive. For AutoDock Vina rescore compounds having score > 1.10 were called active and compounds having scores < 0.42 were called inactive, while in case of Chem rescore, compounds showing scores > 1.03 and scores < 0.28 were considered as active and inactive respectively. The active and inactive compounds, in case of ASP rescore exhibited scores > 0.79 and score < 0.17 respectively, and in case of GOLD rescore active compounds showed scores > 0.7 and inactive compounds had scores < 0.24. In case of consensus rescore compounds having scores > 2.23 were considered as active and compounds having scores < 1.15 were considered inactive (Table 4).

**Table 4:** Efficiency of docking scores and rescores in prediction of training set compounds as active, moderately active and inactive.

	Scoring method	Dataset validated	Most active	Moderately active	Least active
**Score**	**AutoDock Vina**	Most Active	5	3	-
	**AutoDock Vina**	Least active	1	4	4
	**Chem**	Most active	6	2	-
	**Chem**	Least active	1	6	2
	**ASP**	Most active	5	2	1
	**ASP**	Least active	2	2	5
	**GOLD**	Most active	4	3	1
	**GOLD**	Least active	1	4	4
	**Consensus**	Most active	5	3	-
	**Consensus**	Least active	1	4	4
**Rescore**	**AutoDock Vina**	Most active	6	2	-
	**AutoDock Vina**	Least active	-	5	4
	**Chem**	Most active	8	-	-
	**Chem**	Least active	-	5	4
	**ASP**	Most active	5	3	-
	**ASP**	Least active	1	1	7
	**GOLD**	Most active	7	1	-
	**GOLD**	Least active	-	2	7
	**Consensus**	Most active	7	1	-
	**Consensus**	Least active	1	4	4

The results have shown that the efficiency of the docking programs clearly increases when molecular descriptor terms LogP and Nrotb are included to explain ΔG_solv_ and ΔG_conf_ respectively. In the case of GOLD it was observed that only 4 compounds out of the active 8 are in top 8 but in the GOLD rescore the number increased to 7. In Goldscore, one of the active molecules was predicted to be least active, but in the case of GOLD rescore none of the active compounds were predicted as inactive, further substantiating the better efficiency of the rescores (Table 4). Similar results were found in the case of AutoDock Vina, Chem scores and consensus scoring. AutoDock score predicted 5 compounds out of 8 as active and 3 as moderately active, while in case of least active compounds, 4 were predicted as inactive, 4 as moderately active and 1 as active. The results were however found much improved in AutoDock rescore where 6 compounds were predicted as active and 2 as moderately active.

However the best results were found in case of Chem and Gold rescores. Chem rescore predicted all the 8 active compounds as active and 4 out of 9 inactive compounds accurately. While the Gold rescore predicted 7 out of 8 compounds as active and 7 out of 9 compounds as inactive. The results indicate that the rescoring method may be effective in eradicating false positives and false negatives and increasing the accuracy of results. The results indicate clearly that the rescore is predicting the binding affinities of mPGES-1 inhibitors more accurately and the effectiveness of LogP and Nrotb may be further validated on other drug targets and membrane proteins.

Further, the rescoring method was validated using external test set of 100 compounds. Regression analysis was performed against scores obtained for training set of compounds. Each of the scores of different docking programs, consensus scores and the molecular descriptors were further used for the prediction of activity of test set compounds.

The formula used for prediction of pIC_50_ for the test set compounds after regression analysis was:

$$\text{Pr}edicted\;pIC_{50}=w1*Docking\;score+w2*LogP+w3*Nrotb+c$$

Where w1, w2 and w3 = weights obtained after regression analysis on the training set compounds,

Docking score = docking scores of the test set compounds,

LogP = LogP values of test set compounds,

Nrotb = no. of rotatable bonds of test set compounds,

c = intercept obtained from regression analysis.

**Fig 3:**
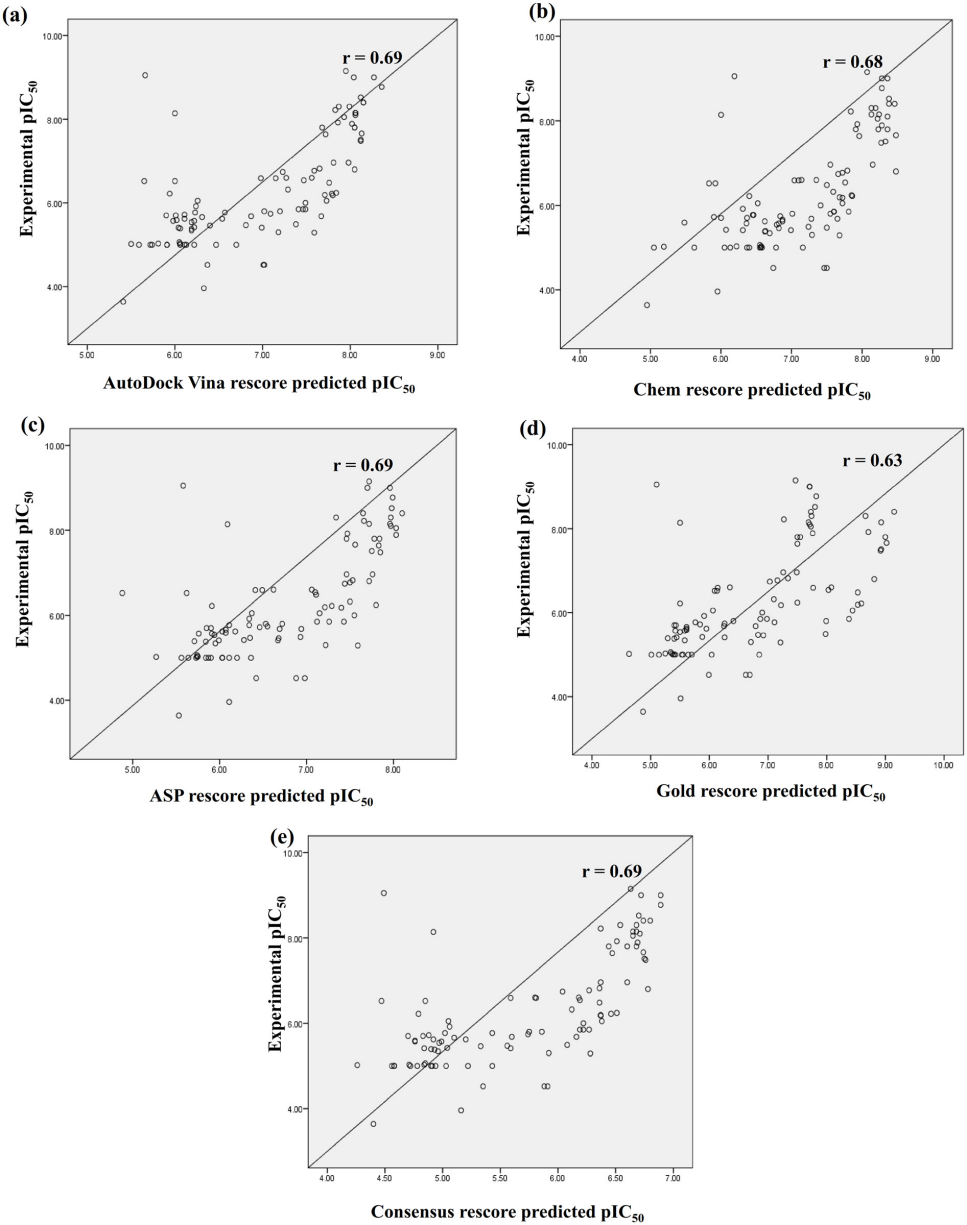
Scatter plots showing coefficient of correlation (r) between the experimental pIC_50_and predicted pIC_50_ by (a) AutoDock Vina rescore, (b) Chem rescore, (c) ASP rescore, (d) Gold rescore and (e) Consensus rescore; for test set compounds.

The correlation coefficient between the experimental and predicted pIC_50_ values for the test set compounds was calculated (Table 5). AutoDock Vina showed the best results where the experimental and predicted pIC_50_ of the test set exhibited the highest correlation (r = 0.70), followed by consensus rescore and ASP rescore (r = 0.69), then Chem rescore (r = 0.68) and Gold rescore (r = 0.63) (Fig 3).

**Table 5:** Pearson and Spearman correlation and sigma 2 tailed tests between predicted pIC_50_ and experimental pIC_50_ of the test set.

		Experimental pIC_50_	Predicted pIC_50_ (Gold rescore)	Predicted pIC_50_ (AutoDock Vina rescore)	Predicted pIC_50_ (ASP rescore)	Predicted pIC_50_ (Chem rescore)	Predicted pIC_50_ (Consensus rescore)
**Experimental pIC**_**50**_	Pearson Correlation	1	.63**	.70**	.69**	.68**	.69**
	Spearman Correlation	1.00	.69**	.72**	.71**	.70**	.72**

** denotes that the correlation is significant at 99% confidence level

When compared to other prominent drug targets from arachidonic acid metabolism, the number of mPGES-1 inhibitors reported is limited. The test set molecules applied for validation of the scoring method contained analogues from various classes of inhibitors with varied activity. It is a well-known fact that the accurate prediction of binding affinities for analogues of the same compounds is a major challenge for many of the existing docking programs. In the present study the high correlation obtained for experimental and predicted pIC_50_ values for the test set compounds validates the efficiency of the scoring method. The results clearly indicate robustness of the developed rescore as it holds well for the external test set compounds.

Current focus of researchers working in the area of molecular modelling and drug design is towards improving the docking scores for the accurate and rapid prediction of binding affinities of inhibitors towards drug targets. The concept of consensus scoring was introduced by Charifson *et al*. [53], wherein the efficiency of various docking programs was evaluated in combinations. They observed that consensus scoring reduced the number of false positives predicted by individual scoring functions, leading to enhancement in number of hits. Even in the present study it was observed that the correlation of docking programs show improvement in consensus as well as rescoring methods. In a number of studies researchers have included some molecular descriptors along with the docking scores to predict the binding affinities accurately. Li *et al*. [54] have developed a new scoring function called ID-Score to assess protein-ligand binding affinities. Their scoring function is based on a comprehensive set of molecular descriptors playing crucial role in protein-ligand interactions. The present study supports previous reports and also hint that molecular descriptors like LogP and Nrotb can be applied as terms for ΔG_sol_ and ΔG_conf_ and may increase the correlation of the docking programs against mPGES-1 inhibitor activity prediction. The results clearly show that the molecular terms considered have an additive effect in the predictions and contribute to the reduction in number of false positives and improvement in prediction of true negatives. The contributions of LogP and Nrotb in the enrichment of binding affinity predictions observed in the present study is in agreement with the reports of Wang *et al*., [55] and ID score of Meng *et al*., [56]. The validation of the approach using an external test set further supports the potential of the scoring method in virtual screening experiments.

The prominent role of LogP in enhancement may be due to the fact that mPGES-1 is a membrane protein and hydrophobicity of ligands helps in efficient transportation to the binding site embedded in the membrane. This hypothesis may be further validated by investigating the effects of LogP on the *in silico* predictions of other membrane proteins. The study may be of significance as there is a need to develop/ improve *in silico* prediction methods that can be applied for mPGES-1 inhibitor activity prediction. There are several reports where the researchers performed docking studies on mPGES-1 to understand its SAR with the inhibitors identified, but there are fewer reports of it being successfully applied for virtual screening procedure for the identification of lead compounds, the main challenge being the limitations of the docking programs. Even though there are few reports of virtual screening methods against mPGES-1, the inhibitors identified showed moderate activity in μM range [57]. As accurate and efficient free energy calculations are time consuming and require high expertise, the rescoring method developed in the present study may be helpful for medicinal chemists in bringing down the time and costs involved in inhibitor development.

## Conclusion

For the identification of inhibitors against drug targets, docking has become an established technique in drug discovery. There are a number of docking programs available but none of them are suitable for all classes of drug targets. This led to the evolution of tuned scoring functions and other rescoring approaches for improvement in the prediction of binding affinities of small molecules towards the drug targets. In this paper, we developed a rescoring method by incorporating the molecular descriptors to explain desolvation penalty and conformational energy of ligands. The rescoring method showed significant improvement in the predictions of training set. The rescore was also effective in differentiating active and inactive mPGES-1 inhibitors in the training set in comparison with the individual docking scores. Further the effectiveness of molecular descriptors LogP and Nrotb was validated using external test set molecules. The results clearly indicate the applicability of LogP and Nrotb in *in silico* mPGES-1 activity prediction.

## Supporting Information

S1 File Supporting Information. Structure and IC50 of the test set compounds (Comp28-Comp69) (Fig A). Structure and IC50 of the test set compounds (Comp70-Comp111) (Fig B). Structure and IC50 of the test set compounds (Comp112-Comp127) (Fig C). Normalized scores of various docking programs and molecular descriptors for the test set compounds (Table A). Experimental and predicted pIC50 values of the rescores for the test set compounds (Table B). The supplementary material can be accessed using the following doi: http://dx.doi.org/10.7910/DVN/IYA8Y6.
(DOC)
