# Supplementary material for: The Augmenting Effects of Desolvation and Conformational Energy Terms on the Predictions of Docking Programs against mPGES-1
Source: PLoS One. 2015 Aug 25;10(8):e0134472. doi: 10.1371/journal.pone.0134472 (PMC4549307; doi:10.1371/journal.pone.0134472)
Supplement: S1 File — Structure and IC50 of the test set compounds (Comp28-Comp69) (Fig A). Structure and IC50 of the test set compounds (Comp70-Comp111) (Fig B). Structure and IC50 of the test set compounds (Comp112-Comp127) (Fig C). Normalized scores of various docking programs and molecular descriptors for the test set compounds (Table A). Experimental and predicted pIC50 values of the rescores for the test set compounds (Table B). The supplementary material can be accessed using the following doi: http://dx.doi.org/10.7910/DVN/IYA8Y6. (DOC) [file pone.0134472.s001.doc]

**Fig a:** Structure and IC_50_ of the test set compounds (Comp28-Comp69)


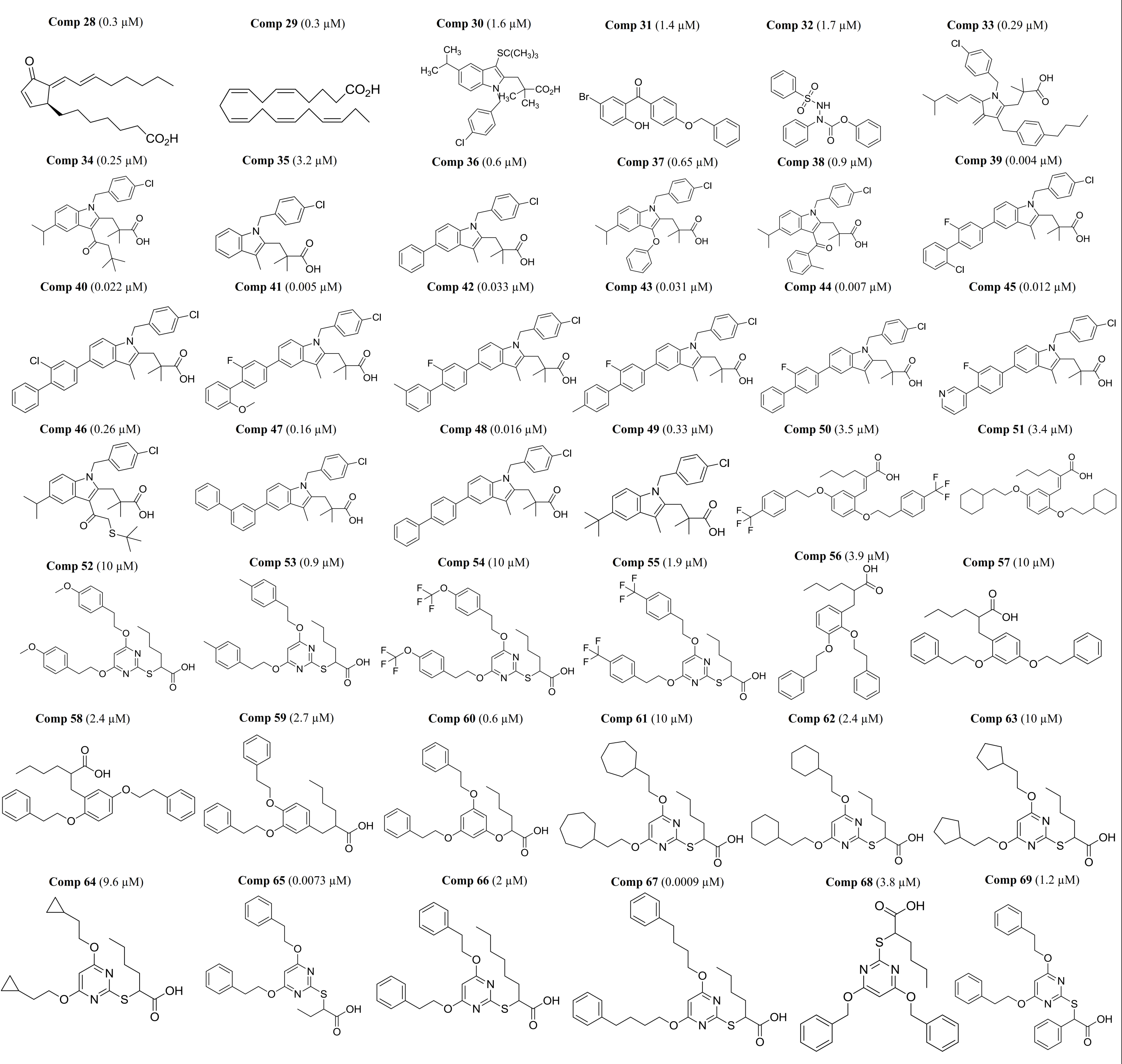


**Fig b:** Structure and IC_50_ of the test set compounds (Comp70-Comp111)


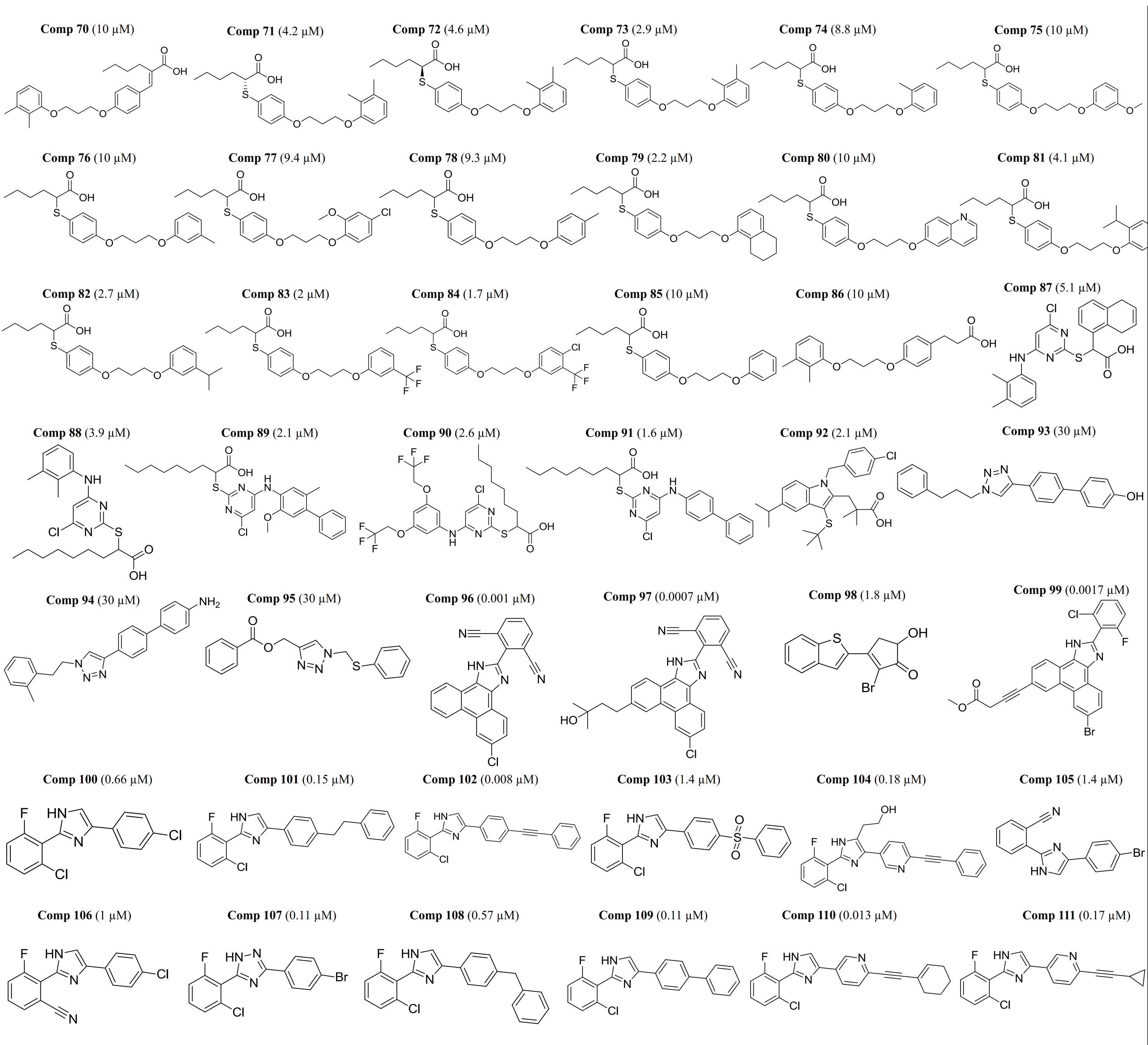


**Fig c:** Structure and IC_50_ of the test set compounds (Comp112-Comp127)


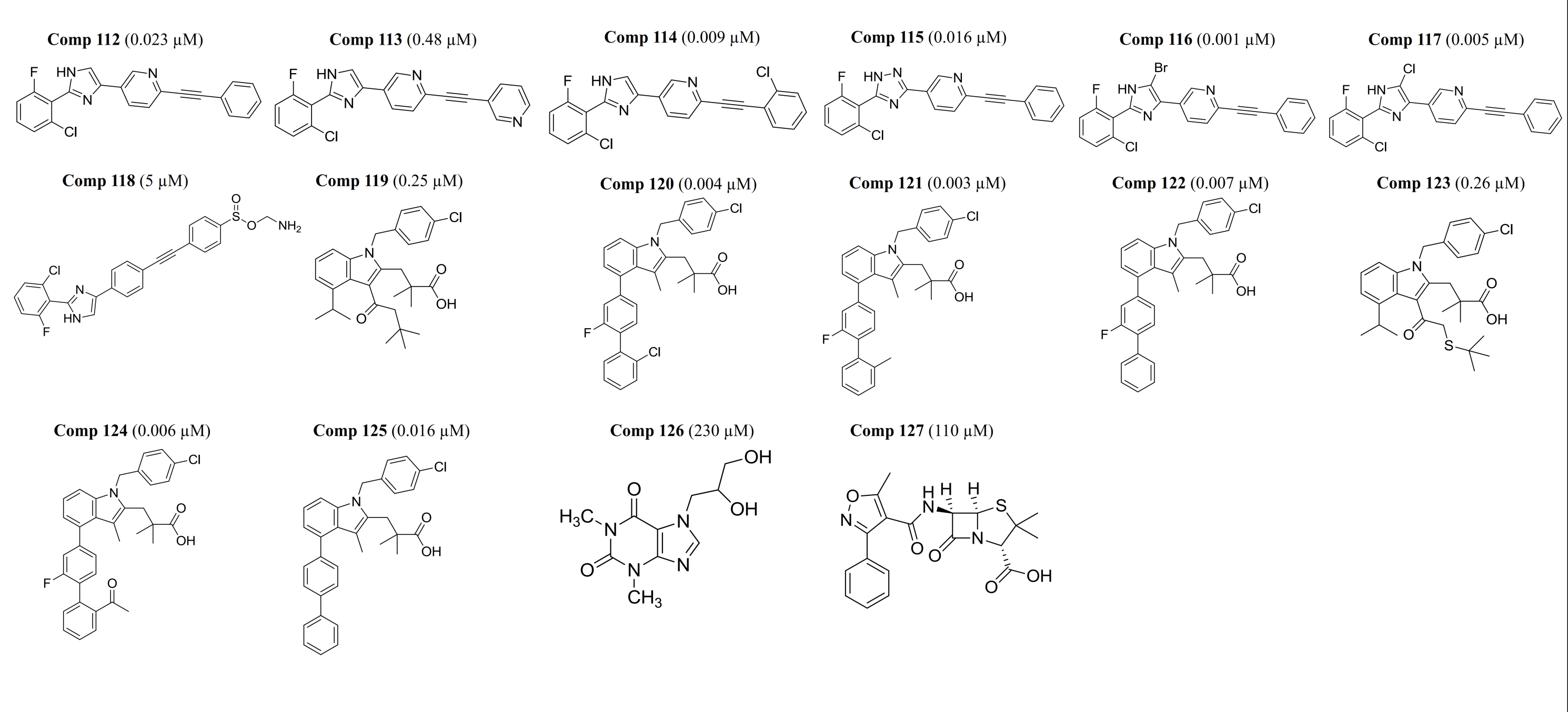


**Table A.** Normalized scores of various docking programs and molecular descriptors for the test set compounds

| **Compounds** | **pIC_50_** | **Auto**  **Dock Vina**  **Score** | **Chem Score** | **ASP Score** | **GOLD Score** | **Consensus score** | **LogP** | **Nrotb** |
| --- | --- | --- | --- | --- | --- | --- | --- | --- |
| 28 | 6.52 | 0.45 | 0.44 | 0.29 | 0.51 | 0.42 | 0.62 | 0.59 |
| 29 | 6.52 | 0.00 | 0.59 | 0.08 | 0.65 | 0.33 | 0.64 | 0.71 |
| 30 | 5.8 | 0.35 | 0.43 | 0.17 | 0.70 | 0.41 | 0.79 | 0.41 |
| 31 | 5.85 | 0.68 | 0.85 | 0.41 | 0.72 | 0.66 | 0.68 | 0.24 |
| 32 | 5.77 | 0.48 | 0.46 | 0.29 | 0.58 | 0.45 | 0.50 | 0.29 |
| 33 | 6.54 | 0.58 | 0.76 | 0.42 | 0.62 | 0.60 | 0.92 | 0.47 |
| 34 | 6.6 | 0.48 | 0.44 | 0.32 | 0.59 | 0.46 | 0.89 | 0.41 |
| 35 | 5.49 | 0.45 | 0.47 | 0.22 | 0.58 | 0.43 | 0.67 | 0.24 |
| 36 | 6.22 | 0.58 | 0.63 | 0.28 | 0.66 | 0.54 | 0.84 | 0.29 |
| 37 | 6.19 | 0.61 | 0.56 | 0.34 | 0.70 | 0.56 | 0.93 | 0.41 |
| 38 | 6.05 | 0.65 | 0.58 | 0.29 | 0.66 | 0.54 | 0.94 | 0.41 |
| 39 | 8.4 | 0.65 | 0.81 | 0.36 | 0.75 | 0.64 | 1.00 | 0.35 |
| 40 | 7.66 | 0.45 | 0.84 | 0.31 | 0.71 | 0.58 | 1.00 | 0.35 |
| 41 | 8.3 | 0.68 | 0.77 | 0.34 | 0.69 | 0.62 | 0.98 | 0.41 |
| 42 | 7.48 | 0.58 | 0.71 | 0.49 | 0.67 | 0.61 | 0.99 | 0.35 |
| 43 | 7.51 | 0.52 | 0.74 | 0.43 | 0.68 | 0.59 | 0.99 | 0.35 |
| 44 | 8.15 | 0.48 | 0.65 | 0.44 | 0.70 | 0.57 | 0.98 | 0.35 |
| 45 | 7.92 | 0.48 | 0.64 | 0.39 | 0.70 | 0.55 | 0.92 | 0.35 |
| 46 | 6.59 | 0.45 | 0.47 | 0.24 | 0.66 | 0.46 | 0.89 | 0.53 |
| 47 | 6.8 | 0.87 | 0.90 | 0.45 | 0.66 | 0.72 | 0.97 | 0.35 |
| 48 | 7.8 | 0.48 | 0.81 | 0.29 | 0.73 | 0.58 | 0.97 | 0.35 |
| 49 | 6.48 | 0.45 | 0.41 | 0.19 | 0.65 | 0.43 | 0.83 | 0.29 |
| 50 | 5.46 | 0.68 | 0.79 | 0.87 | 0.71 | 0.76 | 0.96 | 0.82 |
| 51 | 5.47 | 0.35 | 0.99 | 0.41 | 0.49 | 0.56 | 0.97 | 0.71 |
| 52 | 5 | 0.35 | 0.49 | 0.72 | 1.00 | 0.64 | 0.79 | 0.88 |
| 53 | 6.05 | 0.16 | 0.68 | 0.72 | 0.43 | 0.50 | 0.87 | 0.76 |
| 54 | 5 | 0.58 | 0.00 | 1.00 | 0.53 | 0.53 | 0.95 | 1.00 |
| 55 | 5.72 | 0.23 | 0.34 | 0.91 | 0.43 | 0.48 | 0.94 | 0.88 |
| 56 | 5.41 | 0.26 | 0.67 | 0.60 | 0.29 | 0.45 | 0.81 | 0.76 |
| 57 | 5 | 0.13 | 0.78 | 0.59 | 0.30 | 0.45 | 0.83 | 0.76 |
| 58 | 5.62 | 0.00 | 1.00 | 0.58 | 0.32 | 0.48 | 0.83 | 0.76 |
| 59 | 5.57 | 0.10 | 0.74 | 0.49 | 0.30 | 0.41 | 0.79 | 0.76 |
| 60 | 6.22 | 0.19 | 0.79 | 0.59 | 0.34 | 0.48 | 0.78 | 0.76 |
| 61 | 5 | 0.81 | 0.58 | 0.51 | 0.26 | 0.54 | 0.99 | 0.76 |
| 62 | 5.62 | 0.26 | 0.57 | 0.48 | 0.29 | 0.40 | 0.95 | 0.76 |
| 63 | 5 | 0.65 | 0.44 | 0.49 | 0.25 | 0.45 | 0.83 | 0.76 |
| 64 | 5.02 | 0.13 | 0.26 | 0.42 | 0.19 | 0.25 | 0.65 | 0.76 |
| 65 | 8.14 | 0.77 | 0.50 | 0.61 | 0.30 | 0.55 | 0.68 | 0.65 |
| 66 | 5.7 | 0.32 | 0.55 | 0.65 | 0.37 | 0.47 | 0.88 | 0.88 |
| 67 | 9.05 | 0.13 | 0.82 | 0.67 | 0.36 | 0.50 | 0.93 | 1.00 |
| 68 | 5.42 | 0.52 | 0.40 | 0.62 | 0.35 | 0.47 | 0.74 | 0.65 |
| 69 | 5.92 | 0.35 | 0.55 | 0.64 | 0.36 | 0.48 | 0.75 | 0.65 |
| 70 | 5 | 0.13 | 0.98 | 0.36 | 0.17 | 0.41 | 0.76 | 0.59 |
| 71 | 5.38 | 0.03 | 0.79 | 0.37 | 0.19 | 0.35 | 0.73 | 0.65 |
| 72 | 5.34 | 0.06 | 0.84 | 0.43 | 0.25 | 0.40 | 0.73 | 0.65 |
| 73 | 5.54 | 0.06 | 0.91 | 0.43 | 0.22 | 0.41 | 0.73 | 0.65 |
| 74 | 5.06 | 0.13 | 0.83 | 0.38 | 0.22 | 0.39 | 0.70 | 0.65 |
| 75 | 5 | 0.06 | 0.75 | 0.46 | 0.23 | 0.38 | 0.66 | 0.71 |
| 76 | 5 | 0.52 | 0.83 | 0.47 | 0.23 | 0.51 | 0.70 | 0.65 |
| 77 | 5.03 | 0.42 | 0.76 | 0.53 | 0.29 | 0.50 | 0.68 | 0.71 |
| 78 | 5.03 | 0.03 | 0.83 | 0.38 | 0.22 | 0.37 | 0.70 | 0.65 |
| 79 | 5.66 | 0.23 | 0.89 | 0.45 | 0.22 | 0.44 | 0.77 | 0.65 |
| 80 | 5 | 0.19 | 0.80 | 0.51 | 0.29 | 0.45 | 0.65 | 0.65 |
| 81 | 5.39 | 0.48 | 0.88 | 0.39 | 0.21 | 0.49 | 0.75 | 0.71 |
| 82 | 5.57 | 0.13 | 0.90 | 0.43 | 0.26 | 0.43 | 0.80 | 0.71 |
| 83 | 5.7 | 0.23 | 0.73 | 0.52 | 0.27 | 0.44 | 0.74 | 0.71 |
| 84 | 5.77 | 0.32 | 0.65 | 0.55 | 0.32 | 0.46 | 0.80 | 0.71 |
| 85 | 5 | 0.10 | 0.81 | 0.38 | 0.20 | 0.37 | 0.66 | 0.65 |
| 86 | 5 | 0.19 | 0.75 | 0.37 | 0.18 | 0.37 | 0.57 | 0.47 |
| 87 | 5.29 | 0.58 | 0.64 | 0.55 | 0.23 | 0.50 | 0.79 | 0.29 |
| 88 | 5.41 | 0.23 | 0.54 | 0.45 | 0.18 | 0.35 | 0.90 | 0.59 |
| 89 | 5.68 | 0.58 | 0.81 | 0.60 | 0.26 | 0.56 | 0.98 | 0.71 |
| 90 | 5.59 | 0.52 | 0.12 | 0.76 | 0.40 | 0.45 | 0.97 | 0.94 |
| 91 | 5.8 | 0.65 | 0.91 | 0.50 | 0.25 | 0.58 | 0.97 | 0.65 |
| 92 | 5.68 | 0.39 | 0.57 | 0.19 | 0.08 | 0.31 | 0.92 | 0.41 |
| 93 | 4.52 | 0.90 | 0.87 | 0.41 | 0.24 | 0.60 | 0.62 | 0.29 |
| 94 | 4.52 | 0.87 | 0.88 | 0.43 | 0.25 | 0.61 | 0.56 | 0.24 |
| 95 | 4.52 | 0.90 | 0.78 | 0.47 | 0.26 | 0.60 | 0.50 | 0.35 |
| 96 | 9 | 1.00 | 0.64 | 0.14 | 0.09 | 0.47 | 0.69 | 0.00 |
| 97 | 9.15 | 0.90 | 0.69 | 0.40 | 0.17 | 0.54 | 0.77 | 0.18 |
| 98 | 5.74 | 0.29 | 0.39 | 0.00 | 0.00 | 0.17 | 0.36 | 0.00 |
| 99 | 8.77 | 0.68 | 0.57 | 0.33 | 0.12 | 0.43 | 0.83 | 0.12 |
| 100 | 6.18 | 0.32 | 0.54 | 0.21 | 0.10 | 0.29 | 0.62 | 0.06 |
| 101 | 6.82 | 0.74 | 0.69 | 0.46 | 0.25 | 0.53 | 0.74 | 0.24 |
| 102 | 8.1 | 0.45 | 0.81 | 0.43 | 0.18 | 0.47 | 0.69 | 0.06 |
| 103 | 5.85 | 0.77 | 0.68 | 0.46 | 0.17 | 0.52 | 0.63 | 0.18 |
| 104 | 6.74 | 0.35 | 0.85 | 0.57 | 0.27 | 0.51 | 0.57 | 0.18 |
| 105 | 5.85 | 0.58 | 0.66 | 0.19 | 0.09 | 0.38 | 0.53 | 0.06 |
| 106 | 6 | 0.61 | 0.53 | 0.45 | 0.10 | 0.42 | 0.53 | 0.06 |
| 107 | 6.96 | 0.32 | 0.42 | 0.24 | 0.11 | 0.27 | 0.62 | 0.06 |
| 108 | 6.24 | 0.68 | 0.61 | 0.49 | 0.22 | 0.50 | 0.74 | 0.18 |
| 109 | 6.96 | 0.52 | 0.72 | 0.37 | 0.14 | 0.44 | 0.72 | 0.12 |
| 110 | 7.89 | 0.48 | 0.78 | 0.48 | 0.22 | 0.49 | 0.68 | 0.06 |
| 111 | 6.77 | 0.32 | 0.68 | 0.37 | 0.16 | 0.38 | 0.56 | 0.06 |
| 112 | 7.64 | 0.55 | 0.76 | 0.51 | 0.24 | 0.51 | 0.59 | 0.06 |
| 113 | 6.32 | 0.48 | 0.77 | 0.51 | 0.25 | 0.50 | 0.47 | 0.06 |
| 114 | 8.05 | 0.55 | 0.80 | 0.52 | 0.24 | 0.53 | 0.65 | 0.06 |
| 115 | 7.8 | 0.52 | 0.75 | 0.53 | 0.25 | 0.51 | 0.58 | 0.06 |
| 116 | 9 | 0.58 | 0.83 | 0.43 | 0.19 | 0.51 | 0.68 | 0.06 |
| 117 | 8.3 | 0.58 | 0.75 | 0.46 | 0.22 | 0.50 | 0.67 | 0.06 |
| 118 | 5.3 | 0.39 | 0.64 | 0.49 | 0.19 | 0.43 | 0.61 | 0.24 |
| 119 | 6.6 | 0.32 | 0.47 | 0.22 | 0.08 | 0.27 | 0.87 | 0.47 |
| 120 | 8.4 | 0.65 | 0.76 | 0.63 | 0.23 | 0.57 | 1.00 | 0.35 |
| 121 | 8.52 | 0.52 | 0.78 | 0.57 | 0.27 | 0.53 | 0.99 | 0.35 |
| 122 | 8.15 | 0.55 | 0.72 | 0.58 | 0.25 | 0.53 | 0.98 | 0.35 |
| 123 | 6.59 | 0.32 | 0.62 | 0.27 | 0.13 | 0.34 | 0.84 | 0.53 |
| 124 | 8.22 | 0.32 | 0.59 | 0.54 | 0.19 | 0.41 | 0.97 | 0.41 |
| 125 | 7.8 | 0.48 | 0.72 | 0.48 | 0.20 | 0.47 | 0.97 | 0.35 |
| 126 | 3.64 | 0.32 | 0.17 | 0.26 | 0.15 | 0.23 | 0.00 | 0.12 |
| 127 | 3.96 | 0.65 | 0.27 | 0.20 | 0.06 | 0.30 | 0.31 | 0.18 |

**Table B.** Experimental and predicted pIC_50_ values of the rescores for the test set compounds

| **Compounds** | **Experimental**  **pIC_50_** | **Predicted pIC50**  **(AutoDock Vina**  **rescore)** | **Predicted pIC50**  **(Chem rescore)** | **Predicted pIC50**  **(ASP rescore)** | **Predicted pIC50**  **(GOLD rescore)** | **Predicted pIC50**  **(Consensus rescore)** |
| --- | --- | --- | --- | --- | --- | --- |
| 28 | 6.52 | 6.00 | 5.92 | 5.62 | 6.13 | 4.85 |
| 29 | 6.52 | 5.65 | 5.83 | 4.88 | 6.09 | 4.47 |
| 30 | 5.8 | 7.20 | 7.01 | 6.53 | 7.99 | 5.86 |
| 31 | 5.85 | 7.41 | 7.81 | 7.26 | 8.38 | 6.27 |
| 32 | 5.77 | 6.57 | 6.46 | 6.34 | 7.11 | 5.43 |
| 33 | 6.54 | 7.47 | 7.76 | 7.10 | 8.03 | 6.19 |
| 34 | 6.6 | 7.56 | 7.35 | 7.06 | 8.08 | 6.18 |
| 35 | 5.49 | 7.38 | 7.24 | 6.93 | 7.98 | 6.08 |
| 36 | 6.22 | 7.79 | 7.86 | 7.29 | 8.59 | 6.46 |
| 37 | 6.19 | 7.71 | 7.68 | 7.21 | 8.53 | 6.37 |
| 38 | 6.05 | 7.73 | 7.72 | 7.15 | 8.44 | 6.38 |
| 39 | 8.4 | 8.15 | 8.46 | 7.65 | 9.15 | 6.80 |
| 40 | 7.66 | 8.13 | 8.48 | 7.56 | 9.02 | 6.74 |
| 41 | 8.3 | 7.87 | 8.13 | 7.34 | 8.66 | 6.54 |
| 42 | 7.48 | 8.12 | 8.27 | 7.85 | 8.92 | 6.76 |
| 43 | 7.51 | 8.12 | 8.33 | 7.75 | 8.93 | 6.75 |
| 44 | 8.15 | 8.06 | 8.13 | 7.72 | 8.93 | 6.68 |
| 45 | 7.92 | 7.86 | 7.93 | 7.47 | 8.71 | 6.51 |
| 46 | 6.59 | 7.15 | 7.04 | 6.49 | 7.77 | 5.81 |
| 47 | 6.8 | 8.05 | 8.48 | 7.72 | 8.81 | 6.78 |
| 48 | 7.8 | 8.05 | 8.36 | 7.46 | 9.00 | 6.68 |
| 49 | 6.48 | 7.76 | 7.50 | 7.11 | 8.53 | 6.36 |
| 50 | 5.46 | 6.40 | 6.82 | 6.68 | 6.92 | 5.33 |
| 51 | 5.47 | 6.81 | 7.50 | 6.35 | 6.83 | 5.56 |
| 52 | 5 | 5.59 | 5.62 | 5.73 | 6.85 | 4.58 |
| 53 | 6.05 | 6.26 | 6.52 | 6.37 | 6.06 | 5.05 |
| 54 | 5 | 5.74 | 5.05 | 6.20 | 5.64 | 4.58 |
| 55 | 5.72 | 6.11 | 5.90 | 6.46 | 5.84 | 4.88 |
| 56 | 5.41 | 6.04 | 6.31 | 5.99 | 5.44 | 4.84 |
| 57 | 5 | 6.11 | 6.55 | 6.03 | 5.55 | 4.90 |
| 58 | 5.62 | 6.11 | 6.87 | 6.03 | 5.61 | 4.92 |
| 59 | 5.57 | 5.98 | 6.36 | 5.76 | 5.42 | 4.76 |
| 60 | 6.22 | 5.94 | 6.40 | 5.91 | 5.50 | 4.79 |
| 61 | 5 | 6.70 | 6.78 | 6.36 | 6.04 | 5.43 |
| 62 | 5.62 | 6.54 | 6.62 | 6.18 | 5.95 | 5.20 |
| 63 | 5 | 6.13 | 6.05 | 5.87 | 5.42 | 4.91 |
| 64 | 5.02 | 5.50 | 5.19 | 5.27 | 4.63 | 4.26 |
| 65 | 8.14 | 6.00 | 6.00 | 6.09 | 5.50 | 4.92 |
| 66 | 5.7 | 5.90 | 6.00 | 5.85 | 5.43 | 4.70 |
| 67 | 9.05 | 5.66 | 6.19 | 5.58 | 5.10 | 4.49 |
| 68 | 5.42 | 6.22 | 6.07 | 6.28 | 5.88 | 5.04 |
| 69 | 5.92 | 6.24 | 6.31 | 6.34 | 5.91 | 5.06 |
| 70 | 5 | 6.47 | 7.16 | 6.11 | 5.70 | 5.22 |
| 71 | 5.38 | 6.19 | 6.62 | 5.84 | 5.40 | 4.93 |
| 72 | 5.34 | 6.19 | 6.69 | 5.95 | 5.58 | 4.96 |
| 73 | 5.54 | 6.19 | 6.79 | 5.94 | 5.50 | 4.97 |
| 74 | 5.06 | 6.05 | 6.55 | 5.74 | 5.34 | 4.85 |
| 75 | 5 | 5.72 | 6.13 | 5.56 | 5.01 | 4.56 |
| 76 | 5 | 6.06 | 6.56 | 5.90 | 5.38 | 4.94 |
| 77 | 5.03 | 5.81 | 6.22 | 5.74 | 5.25 | 4.71 |
| 78 | 5.03 | 6.06 | 6.57 | 5.75 | 5.36 | 4.84 |
| 79 | 5.66 | 6.31 | 6.87 | 6.07 | 5.61 | 5.10 |
| 80 | 5 | 5.91 | 6.37 | 5.84 | 5.40 | 4.78 |
| 81 | 5.39 | 6.06 | 6.63 | 5.71 | 5.29 | 4.90 |
| 82 | 5.57 | 6.22 | 6.82 | 5.91 | 5.58 | 4.99 |
| 83 | 5.7 | 6.01 | 6.37 | 5.90 | 5.40 | 4.83 |
| 84 | 5.77 | 6.23 | 6.45 | 6.11 | 5.76 | 5.02 |
| 85 | 5 | 5.91 | 6.40 | 5.64 | 5.14 | 4.72 |
| 86 | 5 | 6.22 | 6.58 | 6.03 | 5.53 | 5.03 |
| 87 | 5.29 | 7.59 | 7.68 | 7.59 | 7.21 | 6.28 |
| 88 | 5.41 | 6.99 | 6.99 | 6.67 | 6.26 | 5.59 |
| 89 | 5.68 | 6.87 | 7.28 | 6.69 | 6.25 | 5.60 |
| 90 | 5.59 | 6.02 | 5.48 | 6.07 | 5.61 | 4.76 |
| 91 | 5.8 | 7.02 | 7.55 | 6.72 | 6.41 | 5.75 |
| 92 | 5.68 | 7.67 | 7.65 | 6.94 | 6.79 | 6.16 |
| 93 | 4.52 | 7.01 | 7.46 | 6.88 | 6.62 | 5.88 |
| 94 | 4.52 | 7.02 | 7.50 | 6.98 | 6.69 | 5.91 |
| 95 | 4.52 | 6.37 | 6.74 | 6.42 | 5.99 | 5.35 |
| 96 | 9 | 8.27 | 8.28 | 7.70 | 7.71 | 6.89 |
| 97 | 9.15 | 7.95 | 8.07 | 7.72 | 7.47 | 6.63 |
| 98 | 5.74 | 7.09 | 6.84 | 6.55 | 6.26 | 5.74 |
| 99 | 8.77 | 8.36 | 8.28 | 7.99 | 7.82 | 6.89 |
| 100 | 6.18 | 7.80 | 7.72 | 7.40 | 7.22 | 6.37 |
| 101 | 6.82 | 7.65 | 7.79 | 7.53 | 7.34 | 6.36 |
| 102 | 8.1 | 8.06 | 8.36 | 7.97 | 7.71 | 6.71 |
| 103 | 5.85 | 7.46 | 7.60 | 7.43 | 6.98 | 6.22 |
| 104 | 6.74 | 7.23 | 7.66 | 7.44 | 7.03 | 6.04 |
| 105 | 5.85 | 7.49 | 7.60 | 7.12 | 6.87 | 6.19 |
| 106 | 6 | 7.49 | 7.41 | 7.55 | 6.90 | 6.22 |
| 107 | 6.96 | 7.81 | 7.55 | 7.46 | 7.26 | 6.37 |
| 108 | 6.24 | 7.84 | 7.85 | 7.80 | 7.50 | 6.51 |
| 109 | 6.96 | 7.98 | 8.15 | 7.76 | 7.49 | 6.60 |
| 110 | 7.89 | 8.02 | 8.28 | 8.03 | 7.76 | 6.69 |
| 111 | 6.77 | 7.59 | 7.72 | 7.50 | 7.16 | 6.27 |
| 112 | 7.64 | 7.72 | 7.96 | 7.83 | 7.50 | 6.47 |
| 113 | 6.32 | 7.29 | 7.59 | 7.50 | 7.10 | 6.12 |
| 114 | 8.05 | 7.93 | 8.22 | 8.03 | 7.73 | 6.65 |
| 115 | 7.8 | 7.68 | 7.91 | 7.84 | 7.50 | 6.44 |
| 116 | 9 | 8.04 | 8.36 | 7.96 | 7.71 | 6.72 |
| 117 | 8.3 | 7.99 | 8.19 | 7.97 | 7.74 | 6.68 |
| 118 | 5.3 | 7.18 | 7.29 | 7.22 | 6.71 | 5.92 |
| 119 | 6.6 | 7.27 | 7.14 | 6.62 | 6.35 | 5.80 |
| 120 | 8.4 | 8.15 | 8.38 | 8.10 | 7.73 | 6.74 |
| 121 | 8.52 | 8.12 | 8.38 | 7.98 | 7.80 | 6.70 |
| 122 | 8.15 | 8.06 | 8.24 | 7.96 | 7.69 | 6.65 |
| 123 | 6.59 | 6.98 | 7.10 | 6.41 | 6.14 | 5.59 |
| 124 | 8.22 | 7.83 | 7.84 | 7.66 | 7.27 | 6.37 |
| 125 | 7.8 | 8.05 | 8.23 | 7.78 | 7.55 | 6.60 |
| 126 | 3.64 | 5.41 | 4.95 | 5.53 | 4.87 | 4.40 |
| 127 | 3.96 | 6.33 | 5.95 | 6.11 | 5.51 | 5.16 |
